# Integrating plant morphological traits with remote-sensed multispectral imageries for accurate corn grain yield prediction

**DOI:** 10.1371/journal.pone.0297027

**Published:** 2024-04-02

**Authors:** Chunhwa Jang, Nictor Namoi, Eric Wolske, Daniel Wasonga, Gevan Behnke, N. Dennis Bowman, D. K. Lee

**Affiliations:** 1 Department of Crop Sciences, University of Illinois at Urbana-Champaign, Urbana, Illinois, United States of America; 2 University of Illinois Extension, Urbana, Illinois, United States of America; Tennessee State University, UNITED STATES

## Abstract

Sustainable crop production requires adequate and efficient management practices to reduce the negative environmental impacts of excessive nitrogen (N) fertilization. Remote sensing has gained traction as a low-cost and time-efficient tool for monitoring and managing cropping systems. In this study, vegetation indices (VIs) obtained from an unmanned aerial vehicle (UAV) were used to detect corn (*Zea mays* L.) response to varying N rates (ranging from 0 to 208 kg N ha^-1^) and fertilizer application methods (liquid urea ammonium nitrate (UAN), urea side-dressing and slow-release fertilizer). Four VIs were evaluated at three different growth stages of corn (V6, R3, and physiological maturity) along with morphological traits including plant height and leaf chlorophyll content (SPAD) to determine their predictive capability for corn yield. Our results show no differences in grain yield (average 13.2 Mg ha^-1^) between furrow-applied slow-release fertilizer at ≥156 kg N ha^-1^ and 208 kg N ha^-1^ side-dressed urea. Early season remote-sensed VIs and morphological data collected at V6 were least effective for grain yield prediction. Moreover, multivariate grain yield prediction was more accurate than univariate. Late-season measurements at the R3 and mature growth stages using a combination of normalized difference vegetation index (NDVI) and green normalized difference vegetation index (GNDVI) in a multilinear regression model showed effective prediction for corn yield. Additionally, a combination of NDVI and normalized difference red edge index (NDRE) in a multi-exponential regression model also demonstrated good prediction capabilities.

## Introduction

Precision agriculture utilizes technology to manage spatial and temporal variability associated with agricultural production to improve crop performance and environmental quality [1, 2]. Combined with nitrogen (N) management, precision agriculture can increase crop productivity and profitability and also enhance environmental quality [1, 3, 4]. Remote sensing (RS) is used to generate high spatial/spectral/radiometric/temporal resolutions required for effective precision agriculture [5]. Some of the applications of RS in agriculture include monitoring crop health over the growing season, predicting crop yield, and determining the schedule, frequency, and quantity of inputs (e.g., application of irrigation water, fertilizer, pesticide, seed, fuel, labor, etc.) [2, 6–8]. Vegetation indices (VIs) are one of the products derived from satellite remote sensing or multispectral sensors mounted on unmanned aerial vehicles (UAVs). Currently, there are over 100 VIs that can be computed from different light spectra, instruments, platforms, and resolutions [9]. The Normalized Difference Vegetation Index (NDVI) is the most common vegetation index used to identify plant vigor levels using red and near-infrared (NIR) wavelengths [10–12]. The Normalized Difference Red Edge (NDRE) uses red and red edge bands and is relatively more suitable than NDVI for intensive crop management because the latter often loses sensitivity after plants accumulate a critical level of leaf cover or chlorophyll content [13–16]. The recently developed Green Normalized Difference Vegetation Index (GNDVI), on the other hand, is efficient for determining N content and estimating corn grain yield [17, 18].

Efficient N application is important for reducing adverse environmental impacts and maximizing crop yields with minimum inputs. Granular urea and liquid urea ammonium nitrate (UAN) are the most popular fertilizers for corn because of their low cost and ease of application. Urea is a highly concentrated nitrogenous fertilizer [19–21] and is readily converted to mobile forms of N through microbial transformations [22]. However, up to 40 − 70% of N is lost because of the high solubility and volatilization of urea [23]. Compared to other formulations, UAN is relatively easy to handle and apply. When injected into the soil, UAN considerably reduces the risk of ammonia volatilization [24]. Typically, N is applied during the non-growing season (e.g., fall, early spring, and pre-plant), at planting, or in-season (e.g., side-dressing) to match crop needs for maximum availability. In general, corn requires little N between vegetative stages and the fifth leaf development stage (V5), but crop N demand increases during the eighth leaf (V8) to tasseling (VT) development stages [25, 26]. Among N application methods, side-dressing is considered appropriate for targeted N application as it allows for adjustments to planned N supply. However, in-season N applications are associated with risks including delayed application due to adverse weather conditions and environmental losses through leaching and volatilization [27]. To overcome this problem, controlled-release fertilizers (CRF) (i.e., slow-release fertilizers) have been shown to enhance N use efficiency, leading to decreased N amounts required to sustain production [28–30].

To achieve sustainable corn production, the selection of an appropriate N source and correct amount are critical. Additionally, continuous monitoring is essential to promptly identify nutrient deficits and poor plant health. Unmanned aerial vehicles combined with effective N application techniques can be critical for achieving the goals of improving corn management and yield while mitigating adverse impacts on the environment [29, 31, 32]. In this study, we aimed to compare various remotely sensed VIs, including NDVI, NDRE and GNDVI for in-season plant vigor and any nutrient deficiencies responses to N application rates and N sources, and to evaluate the effectiveness of VIs for end-season corn yield prediction.

## Materials and methods

### Study site

The field experiment was conducted at the University of Illinois South Farm, Savoy, IL (40° 04’ 34” N, 88° 14’ 46” W) on a Catlin silt loam soil (fine-silty, mixed, superactive, mesic Oxyaquic Argiudolls). Pre-planting soil properties from 0–30 cm depth were pH of 6.6, organic matter of 3.5%, phosphorus (P) of 36.5 ppm, potassium (K) of 110 ppm, and available nitrogen (N) of 13.4 ppm (NO_3_-N) and 3.05 ppm (NH_4_-N). Corn (Kekalb DKC60- 87RIB) was seeded on May 24 of 2021, with the seeding rate of 95000 seeds ha^-1^ in a 0.7621 m row spacing. The plots (3m x 4.6 m) were arranged in a completely randomized block design (CRBD) and replicated four times. Three fertilizer formulations were tested, including liquid urea ammonium nitrate (UAN), granular urea, a slow-release fertilizer, and a control (unfertilized) (Table 1). The controlled-release fertilizer (hereafter referred to as CRF) is a new sigmoid-release type fertilizer (S-Coat) (Nousbo Co., Ltd. Suwon, South Korea), which consists of N 21%, P_2_O_5_ 7%, K_2_O 9%, and MgO 1%. Both UAN and granular urea were applied at 208 kg N ha^-1^ which is the maximum return to nitrogen (MRTN) in central Illinois [33, 34]. The CRF was banded in furrows at 52, 104, 156, and 208 kg-N ha^-1^ rates corresponding to 25%, 50%, 75%, and 100% of the MRTN. Both UAN and granular urea treatment were preceded by starter applications of monoammonium phosphate (MAP) (11-52-0) and muriate of potash (MOP) (0-0-60) applied at 112kg and 183kg rates, respectively. Liquid UAN was broadcasted at the full rate of 208 kg N ha^-1^ while granular urea (hereafter referred to as urea-side) was split-applied with 50% banded at planting and 50% side-dressed at V6-V8 stage.

**Table 1:** Fertilizers treatments, rates used and application strategy in 2021 at Savoy, IL.

Treatment^a^	N rate (kg N ha^-1^)	N strategy^b^
Control	0	Unfertilized
UAN	208	32% UAN
		MAP (11-52-0)
		MOP (0-0-60)
Urea-side	208	46% Urea
		MAP (11-52-0)
		MOP (0-0-60)
CRF-208N	208	CRF S-coat
CRF-156N	156	
CRF-104N	104	
CRF-52N	52	

^a^UAN is liquid urea ammonium nitrate; CRF is controlled-release fertilizers

^b^MAP is monoammonium phosphate; MOP is muriate of potash; S-coat is sigmoid release type fertilizer

### Morphological trait measurement

Emergence count was performed 11 days after sowing (4 June of 2021) on 1 m^2^ area. The plant height and leaf chlorophyll content were measured biweekly using a Leveling Rod and the Soil Plant Analysis Development (SPAD) chlorophyll meter SPAD-502 (Minolta corporation, Ltd., Osaka, Japan) respectively.

### Unmanned aerial vehicle (UAV) image acquisition

Drone flights were conducted every four weeks after corn emergence during the 2021 growing season (i.e., June 25, July 05, August 04, and September 03 of 2021 (Table 2). The flights were performed using a DJI Inspire 2 quadcopter drone, mounted with a nadir-fixed 3.2 MP six-sensor Micasense Altum (Ageagle Sensor Systems Inc., Seattle, WA) with blue (459–491 nm), green (546.5–573.5 nm), red (661–675 nm), red edge (711–723 nm), near-infrared (813.5–870.5 nm), and long-wave infrared thermal (8–14 μm) sensors flown in a single north-south serpentine flight pattern. The immediate onboard GPS of the UAV was recorded into the metadata of each image and was utilized during image reconstruction by the software. Flights were conducted on days with low wind conditions (< 5 meters per second) within two hours of solar noon. Due to the time required to capture all the required photographs, cloud cover varied between missions, but most photographs were collected in either full sun or constant light cloud cover. An onboard Micasense DLS-2 light sensor provided solar directional and intensity measurements during the flight and was written into the metadata of each photograph for calibration pre-processing. Inspire 2 flights were flown to maintain an 80% front and a 75% side overlap for the Altum sensor at 20 m using the DJI Pilot flight mission planning application. The ground sampling distance (GSD) of the orthomosaics was 0.86 cm/pixel. A Micasense calibration panel was recorded before and after each mission with the Altum to aid in image processing.

**Table 2:** UAV flight campaign and morphological trait measurement on different days after emergence (DAE).

Date	Corn growth stage	DAE^a^	Multispectral imaging	Morphological trait measurements
05/24/2021	Planting			
06/04/2021	Emergence	1	×	
07/05/2021	Early growing season (V6)	31	×	
07/06/2021		32		Height/SPAD
07/22/2021	Mid growing season (VT-R3)	48		Height/SPAD
08/04/2021		61	×	
08/11/2021		68		Height/SPAD
08/27/2021	Late growing season (mature)	84		SPAD
09/03/2021		91	×	
10/11/2021	Harvest	129		

^a^DAE is days after emergence

### Image processing

Images from UAV missions were uploaded to Metashape (Agisoft LLC, St. Petersburg, Russia) and converted into a local coordinate system (NAD83(2011) / Illinois East (ft US); EPSG: 6455) and processed using structure from motion photography (SfM) using a high-performance workstation. Images from the Altum were calibrated by masking out the calibration panels and calibrating reflectance in Metashape. Photos were aligned with high accuracy, and ground control points (GCP) were identified within each photo and the coordinates were uploaded to Metashape. Dense point clouds were generated using high-quality and mild depth filtering. Digital elevation models (DEMs) were generated from the dense cloud with high quality and mild depth filtering. The orthomosaic was generated using the DEM surface. The orthomosaic and DEM were exported in a common coordinate reference system (WGS 84/World Mercator; EPSG 3395) in GeoTIFF format without any file compression. Four vegetation indices (VIs) were estimated based on the generated orthomosaics at V6 (31 DAE), R3 (61 DAE), and mature (91 DAE) (Table 3).

**Table 3:** Summary of vegetation indices estimated from multispectral images.

Vegetation index^a^	Formula^b^	Reference
ExG	2G-R-B G = g/(g+r+b), R = r/(g+r+b), B = b/(g+r+b)	[35]
NDVI	(NIR-r)/(NIR+r)	[36]
NDRE	(NIR-RE)/(NIR+RE)	[37]
GNDVI	(NIR-g)/(NIR+g)	[38]

^a^ExG is Excess green; NDVI is Normalized Difference Vegetation Index; NDRE is Normalized Difference Red Edge Index; GNDVI is Green Normalized Difference Vegetation Index

^b^r is red; g is green; b is blue; NIR is near-infrared; RE is red edge

**Fig 1:**
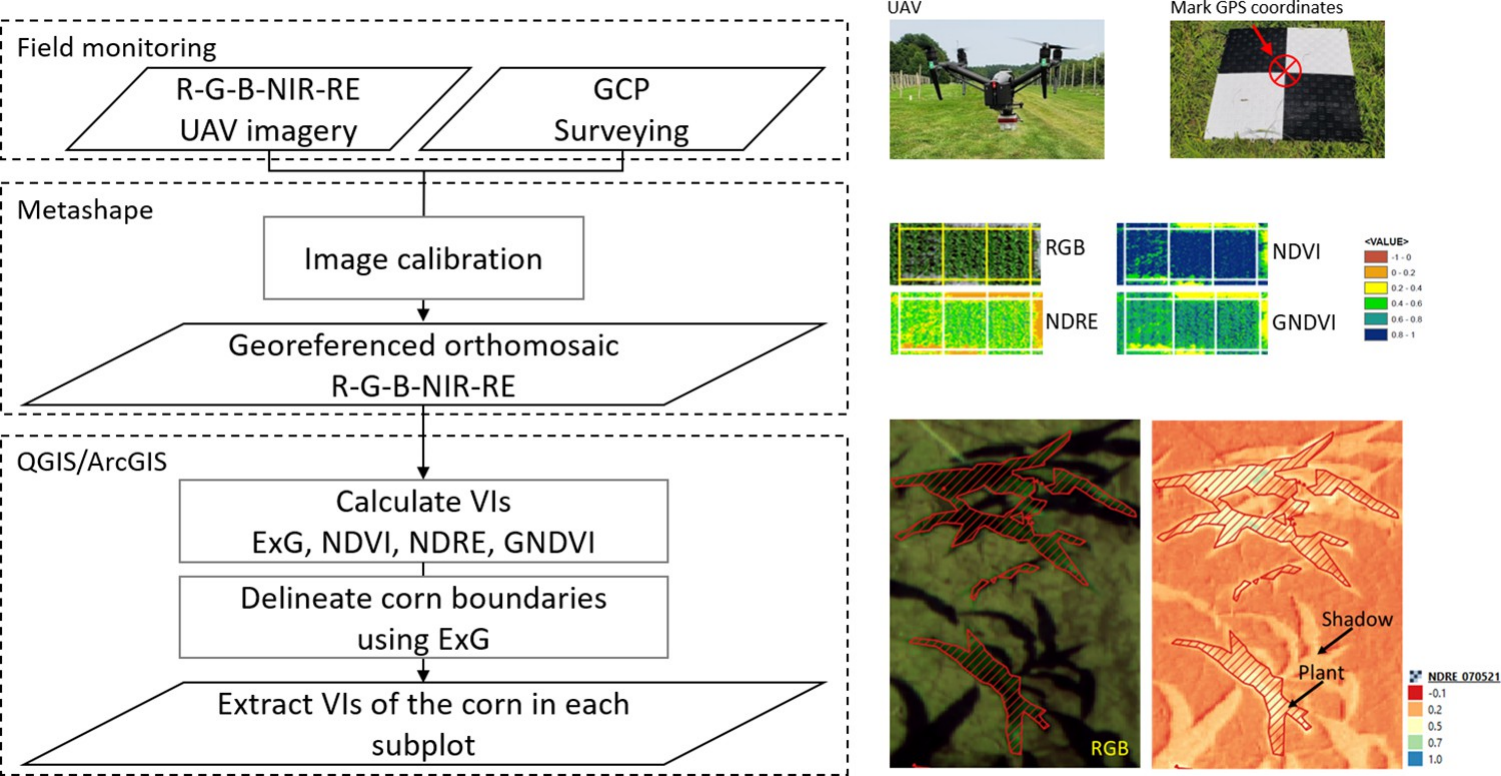
Scheme of the image processing.

The vegetation indices were calculated using the raster calculator in Quantum Geographic Information System (QGIS). The plant canopy cover was estimated from the Excess Green vegetation index (ExG) using the System for Automated Geoscientific Analysis (SAGA) in QGIS. In general, the healthiest crops have a low ExG index value (0–1.5), and the dry crops or barren soil have a high value (> 1.9) [35, 39, 40]. In this study, the threshold was less than 0.75 in the ExG index (0.7–0.8) for plant canopy cover. By classifying pixels into two classes, a binary raster was generated, where pixels with a value of 0 belong to both soil and shadow and pixels with a value of 1 belong to vegetation. The pixel values are replaced by neighboring majority values using the majority filter module. Finally, the raster file was converted into a polygon shapefile using the polygonize module, and the average VIs were extracted from the plants in each subplot using the zonal statistic module in ArcGIS 10.7.1. The remote sensed traits monitored were NDVI, NDRE, and GNDVI. The image processing is summarized in Fig 1.

### Grain yield prediction

The corn was harvested at maturity (129 days after emergence) using ALMACO small plot combine (Almaco Inc., Nevada, IA), and the grain yield was determined based on machine-recorded moisture content of 15%. The coefficient of variation (CV) was calculated to determine the variability of corn yield. Generally, CV<10 is very good, 10–20 is good, 20–30 is acceptable, and CV > 30 is not acceptable [41]. The strength of the correlation between morphological traits (height, SPAD and emergence count) and grain yield was evaluated using Pearson’s correlation (r) in R. Generally, r > 0.69 is a considered high to very high correlation, 0.5–0.69 is a moderate correlation, 0.3–0.49 is a low correlation, and 0–0.29 is interpreted as little if any correlation [42].

### Statistical analysis

Analysis of variance (ANOVA) was conducted to test the effect of treatment (control, UAN, urea-side, CRF-52N, CRF-104N, CRF-156N, and CRF-208N), sampling date, and their interaction on the measured traits. Where significant (*p < 0*.*05*), means were separated using Tukey’s HSD using the package ‘agricolae’ in R [43]. To predict corn grain yield, single linear and exponential regression models were built using morphological and remotely sensed plant traits. Traits were combined using multiple linear and multiple exponential models to determine whether grain yield prediction can be improved by including multiple traits. In both models, the morphological trait measurements were set as response variables while the VIs were set as predictor variables. The sum of squares (SOS) and corrected Akaike information criterion (AICc) were determined to evaluate the best-fit model between linear and exponential regression models using the package ‘stats’ in R [44]. Due to the small dataset, the models were validated using 5-fold cross-validation using the package ‘caret’ in R [45, 46]. Model performance was evaluated using the root mean square error (RMSE), mean absolute error (MAE), and coefficient of determination (R^2^).

## Results and discussion

### In-season morphological trait measurements

The sampling time and treatments showed significant (p < 0.01) effects on the plant height, and SPAD, but no difference on the emergence counts (Table 4). Moreover, there was no interaction effect between sampling time and treatment on the measured parameters.

**Table 4:** Analysis of variance (ANOVA) showing effects of treatment and sampling time and their interactions on morphological trait measurements.

Effect	Emergence count	Height	SPAD
Treatment	0.633	0.001	< 0.001
Sampling time	-	< 0.001	< 0.001
Treatment × Sampling time	-	0.227	0.086

**Fig 2:**
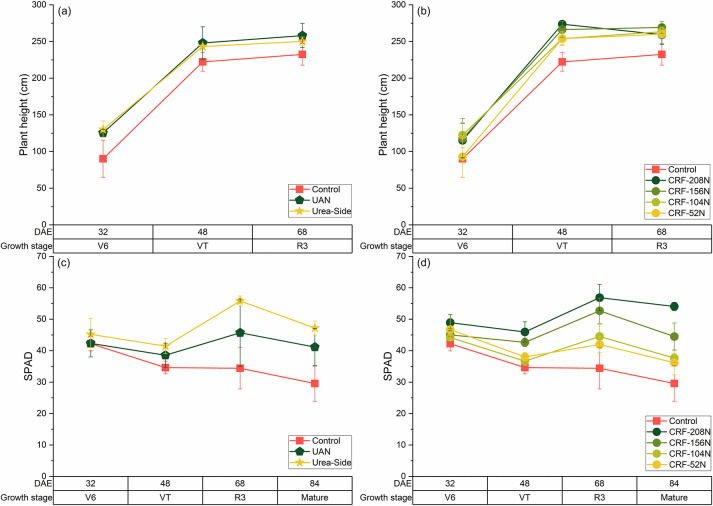
Morphological traits as affected by sampling season, N rate, and fertilizer application treatments. The plant height reached full height at VT under all treatments (a) and (b). There was a clear difference in SPAD reading between the control (0 kg N ha^-1^) and other treatments (c) and (d). Error bars represent the standard deviation.

Across all treatments, the plant height increased rapidly between 32 DAE (V6) and 48 DAE (VT), and then leveled (Fig 2A and 2B). There were windstorms during the R3 corn stage, and minor damage was observed on the relatively taller plants of the CRF-208N plots (Fig 2B). However, plant height did not differ between N rates of the CRF (p > 0.05) after the VT stage (Fig 2B). Unlike, Liu and Wiatrak [47], we found no changes in plant height with fertilizer treatments and rates, suggesting a limitation in discriminating between different N rates in corn. In Liu and Wiatrak [47], the difference in plant height under various N rates was evident due to low soil fertility. In contrast, soil at the beginning of our study had greater residual nutrient concentrations of P (1.3-fold) and K (1.9-fold), and organic matter (2.2-fold). Unlike plant height, SPAD values differed depending on N treatment and rates. The SPAD reading in control plots generally declined over the season (Fig 2C and 2D) but increased with increasing N rates in all the CRF treatments (Fig 2D). SPAD readings have a positive relationship with chlorophyll content and N content per leaf area, and high SPAD readings account for high leaf N status [48].

### Remote sensed vegetation indices for plant stand monitoring during the growing season

**Fig 3:**
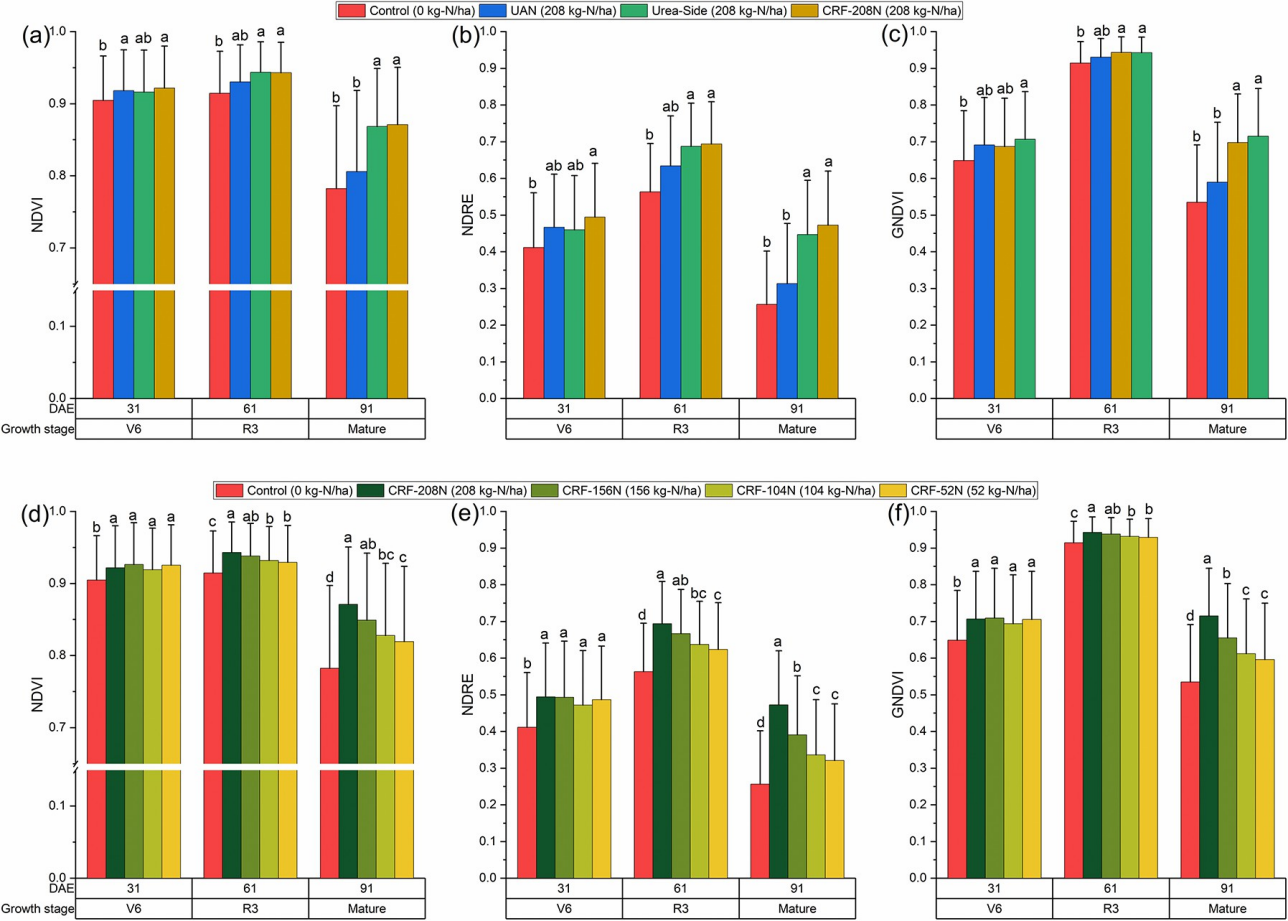
The vegetation indices as affected by N rate and fertilizer application treatments. Vegetation indices differed depending on fertilizer application methods when applying the highest N rate (208 kg N ha-1) (a)-(c). Vegetation indices rose as the N rate increased under CRF treatment (d)-(f). Error bars represent the standard deviation. The statistical labels stem from a mean comparison analysis using the least significant difference (LSD) test (α = 0.05).

The vegetation indices varied depending on the growth stage and N treatments (Fig 3). The remote sensed vegetation indices can be used to identify vegetation and measure its health and vitality. The UAN treatment and the unfertilized control showed no differences on plant health, even though N was applied at the highest level with 208 kg-N ha^-1^ (Fig 3A–3C). However, the urea-side and CRF-208N showed good plant stands at 91 DAE (mature) when compared to UAN (one-time high fertilizer at planting). The urea-side and CRF-208N treatments did not show any differences in NDVI, NDRE, and GNDVI. The peak vegetation indices were at 61 DAE (R3) in NDVI, NDRE, and GNDVI.

Importantly, NDRE detected differences in plant stand between treatments at 61 DAE (Fig 3B and 3E), and earlier than NDVI and GNDVI. In NDVI and GNDVI, similar values were observed during the first two monitoring dates (31 and 61 DAE) between treatments, and only the control showed a clear difference compared with other treatments (Fig 3A, 3C, 3D and 3F). At 91 DAE, clear differences in NDVI and GNDVI between control and UAN vs. urea-side and CRF-208N were noticeable (Fig 3A and 3C). The CRF treatment showed good plant stand at 31 and 61 days after emergence, with more variation occurring following 91 DAE. In addition, N rate within a wide range can be distinguished by VIs. For example, the low fertilization group (CRF-104N and CRF-52N, < 104 kg-N ha^-1^) had similar low vegetation indices over the growing season, suggesting that the low fertilization had reduced corn plant health compared to the high fertilization group (CRF-208N and CRF-156N, > 156 kg-N ha^-1^). The high fertilization group showed significantly high VIs at the mature corn stage (91 DAE), except for NDVI.

In Fig 3, the sharp decline in all VIs (NDVI, NDRE, and GNDVI) after 91 DAE, can be attributed to physiological maturity, change in crop color, and leaf senescence [49]. At the maturity stage, all the VIs captured contrasting differences in corn stand health between the control and other N treatments. However, the sensible degrees were different among VIs, NDVI was the most insensitive compared to NDRE and GNDVI. In our study, NDRE was better at detecting in-season crop response to N fertilization. These results corroborate previous findings that NDRE had strong relationships with N rates, followed by NDVI and GNDVI [53]. Furthermore, Sumner et al. [50] suggested that the simplified canopy chlorophyll content index (SCCCI) (NDRE/NDVI) may have a better relationship with corn tissue N status. These findings show VIs from UAVs can distinguish the N treatment and N rate on corn. This observation concurs with previous findings that NDRE could be used for N management under various crops [50–52].

### Correlation between corn grain yield and variables

**Fig 4:**
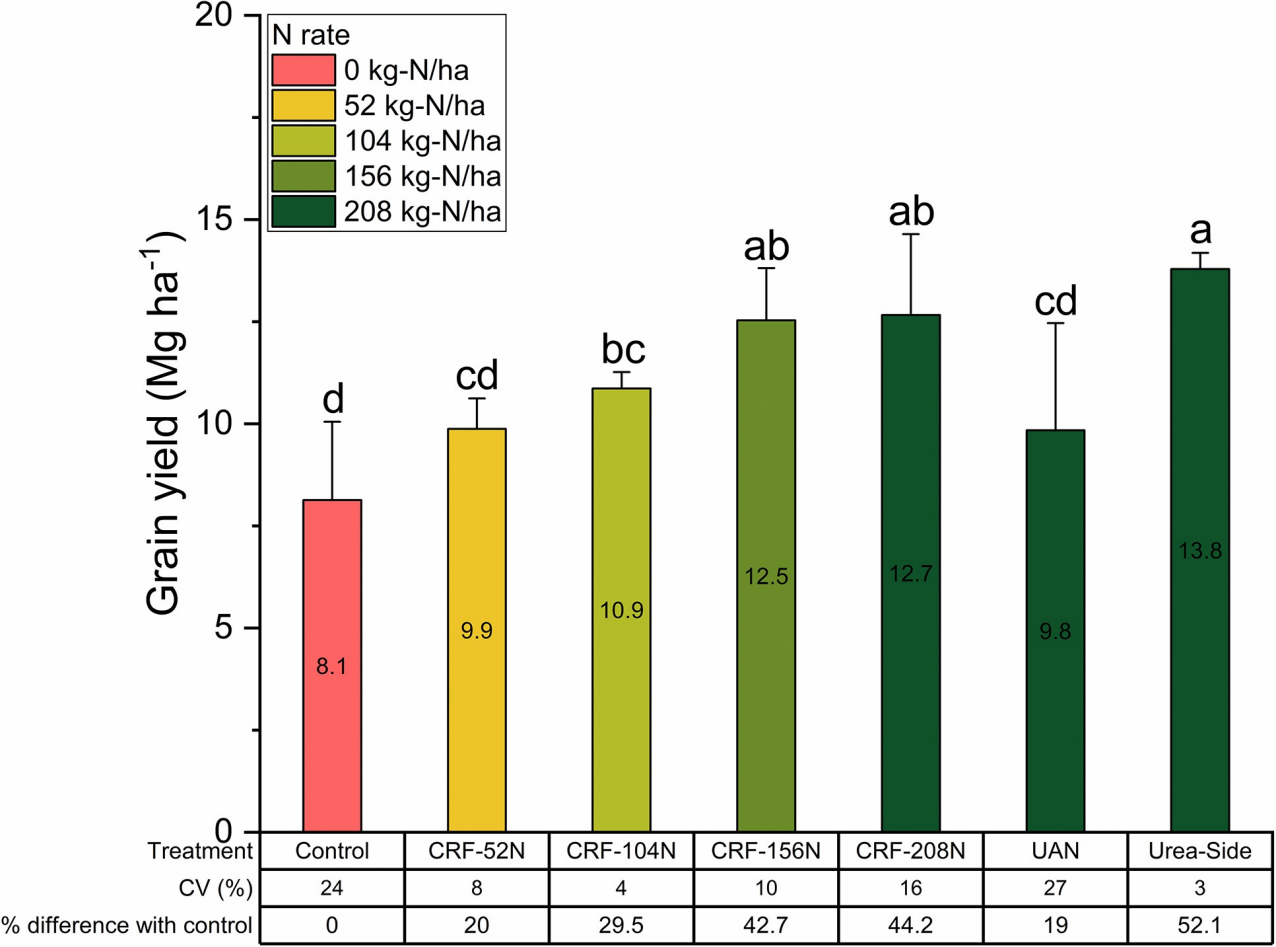
Corn grain yield as affected by N rates and fertilizer application treatments. The fading color from dark green to yellow indicates the highest N rate to the lowest N rate. Error bars represent the standard deviation. The statistical labels stem from a mean comparison analysis using the least significant difference (LSD) test (α = 0.05).

Corn grain yield increased by 29.5 to 52.1% relative to control in the CRF treatments, except for CRF-52N (*p < 0*.*05*) (Fig 4). At the highest N rate of 208 kg-N ha^-1^, only UAN treatment did not exhibit an advantage in yield. Side-dressing urea at 208 kg-N ha^-1^ produced grain yields comparable to CRF-156N and CRF-208N (*p > 0*.*05*). Plant height was correlated moderately with grain yield during the entire growing season (r = 0.44–0.58) (Table 5). This suggests that the plant height might not be a crucial factor in determining the grain yield potential, but the grain yield was affected by the type of N source and N level in our study. In contrast, Yin et al. [53] noted a strengthening of relationship between corn yield and plant height from early to late corn growth stage.

**Table 5:** Pearson correlation (r) between corn grain yield and traits under different sampling season.

Traits	Sampling season		Corn grain yield	
	Days after emergence	Plant growth stage	r	p-value
N rate	-	-	0.61	***
Plant height	32d	V6	0.44	*
	48d	VT	0.58	**
	68d	R3	0.49	**
SPAD	32d	V6	0.43	*
	48d	VT	0.67	***
	68d	R3	0.75	***
	84d	Mature	0.77	***
NDVI	30d	V6	0.51	**
	60d	R3	0.85	***
	90d	Mature	0.88	***
NDRE	30d	V6	0.51	**
	60d	R3	0.86	***
	90d	Mature	0.82	***
GNDVI	30d	V6	0.56	**
	60d	R3	0.86	***
	90d	Mature	0.85	***

***Correlation is significant at the 0.001 level

**Correlation is significant at the 0.01 level

*Correlation is significant at the 0.05 level

The SPAD measurements exhibited weak correlations with grain yield at V6 (Table 5). However, unlike height, the strong association with yield was observed as the season progressed. Except for plant height, all other phenotypic and remote sensed traits significantly correlated with grain yield between 60 and 90 DAE and ranged from 0.75 to 0.88 (*p < 0*.*001*). Moreover, late season VIs had stronger correlation with grain yield (r > 0.82 and *p < 0*.*001*), in line with findings that indicate later season sensor-based data are closer associated with the corn grain yield compared with early season data [47, 54–56].

### Corn yield prediction using exponential and linear regression models

**Fig 5:**
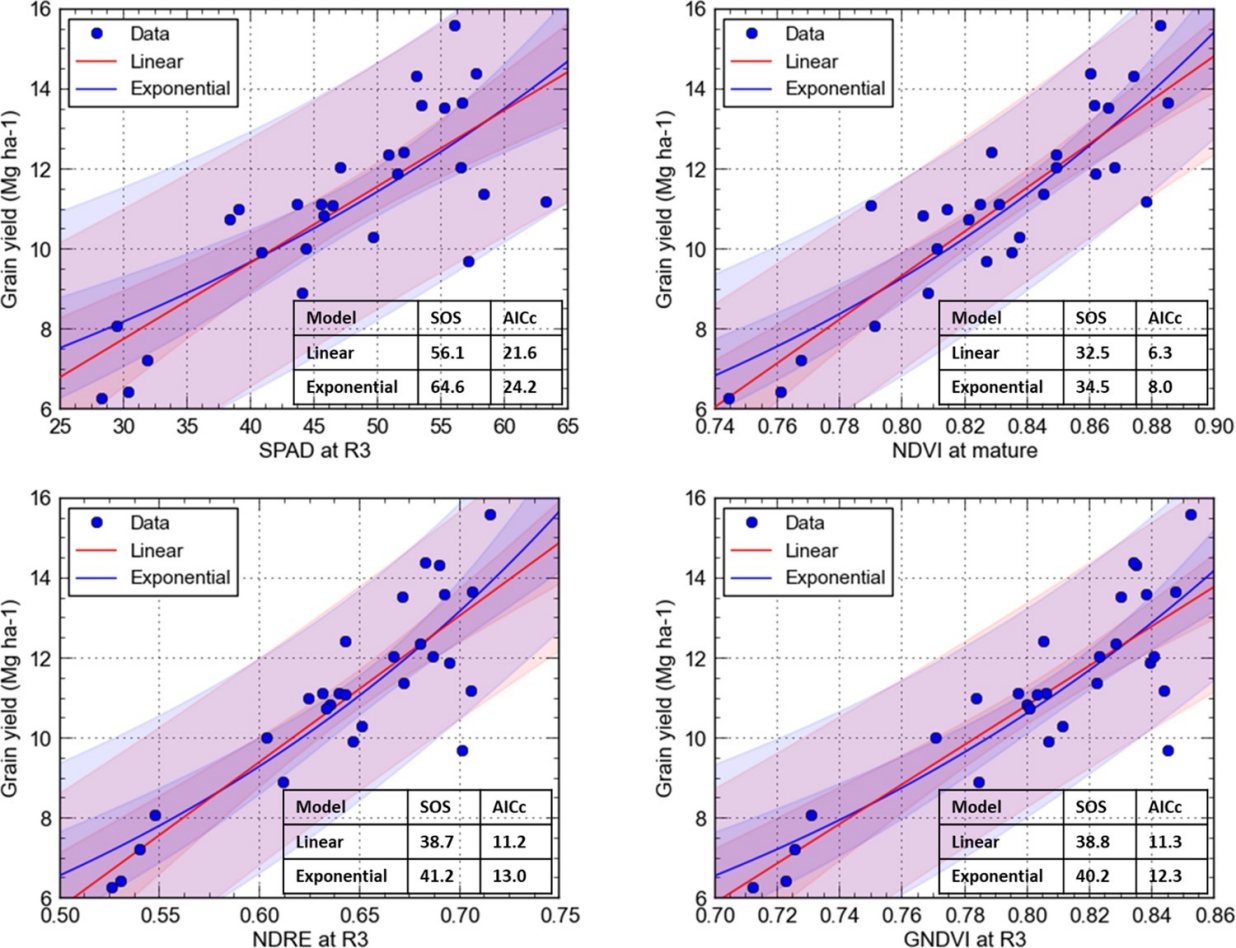
Grain yield was positively correlated with SPAD and vegetation indices at R3 and mature corn growth stage. Blue and red shaded areas indicate the 95% confidence intervals associated with model fit.

**Fig 6:**
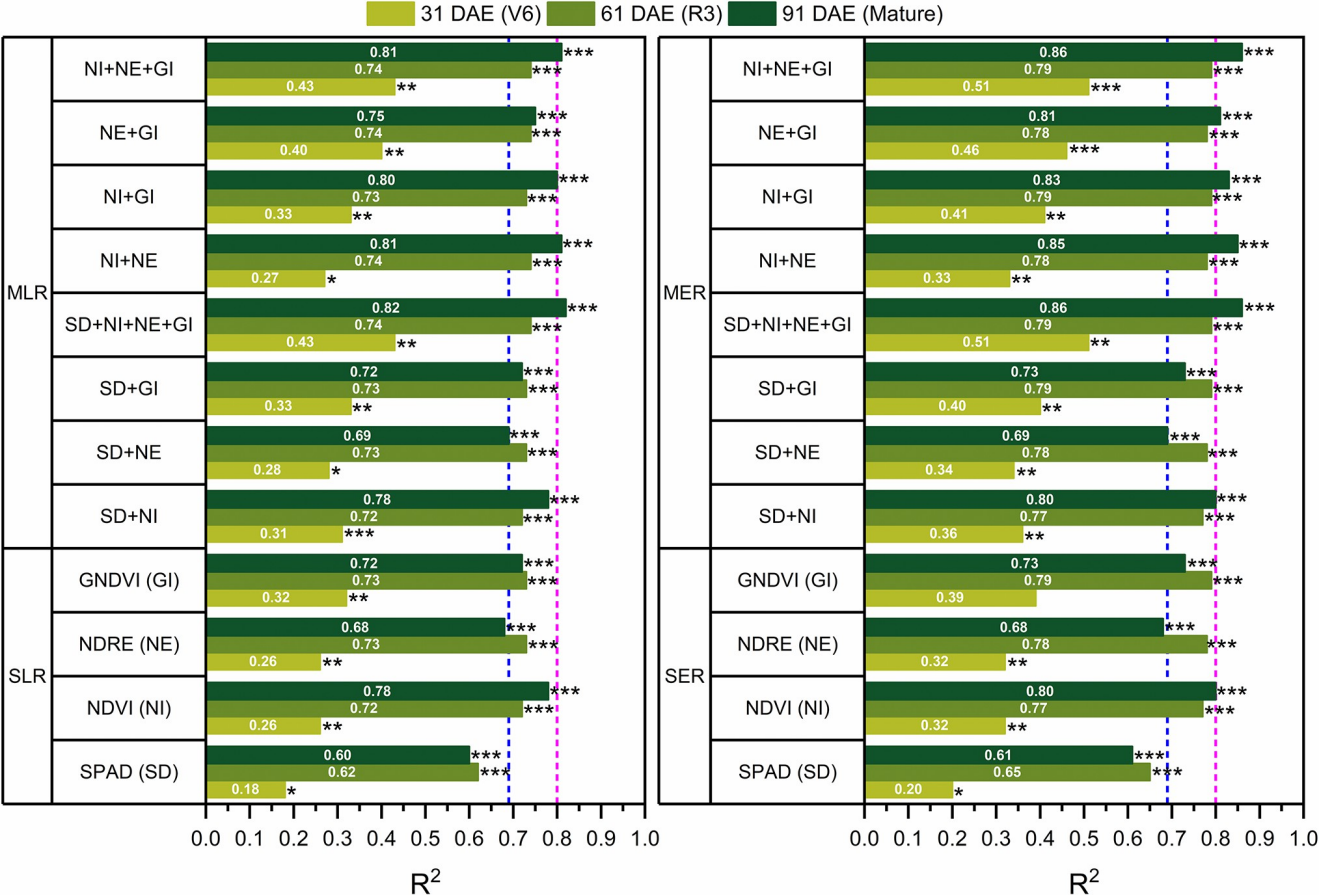
Model performance of simple and multiple regression model (R2). P values less than 0.05 are given one asterisk, P value less than 0.01 are given two asterisks, and P values less than 0.001 are given three asterisks. Blue line indicates R^2^ = 0.69 and pink line indicates R^2^ = 0.8.

Selected results from regression involving traits collected at different stages of the growing season are shown in Fig 5. The linear model had a lower sum of squares (SOS) and corrected Akaike information criterion (AICc) than the exponential model when using single remote sensing derived and morphological traits (Fig 5). Among various traits in, the smallest SOS and AICc were observed in linear (SOS = 32.5 and AICc = 6.3) and exponential (SOS = 34.5 and AICc = 8.0) models using NDVI at the mature stage (90 DAE). Whereas the model performance (R^2^) slightly improved when an exponential regression model as opposed to a linear one (Fig 6 and Table 6). Both linear and exponential regression models using early season predictors (V6) generated weaker predictions of grain yield (R^2^ ≤ 0.51) compared to models that used the middle (R3) and late (mature) season predictors (Fig 6).

**Table 6:** Corn grain yield estimation with predictors under three sampling seasons (V6, R3, and mature of corn). The models were derived based on simple linear regression (SLR), multiple linear regression (MLR), simple exponential regression (SER), and multiple exponential regression (MER).

Model^a^	Predictor(s)	Early (V6)	Middle (R3)	Late (Mature)
SLR	SPAD	Y = 0.23×SPAD+0.97	Y = 0.19×SPAD+2.04	Y = 0.21×SPAD+2.41
	NDVI	Y = 135.89×NDVI-113.79	Y = 158.33×NDVI-136.67	Y = 54.92×NDVI-34.57
	NDRE	Y = 30.32×NDRE-3.14	Y = 36.56×NDRE-12.5	Y = 22.18×NDRE+3.08
	GNDVI	Y = 48.82×GNDVI-22.7	Y = 49.48×GNDVI-28.74	Y = 27.38×GNDVI-6.09
MLR	SPAD+NDVI	Y = 0.13×SPAD+106.47×NDVI-92.34	Y = 0.01×SPAD+152.3×NDVI-131.5	Y = 0.01×SPAD+52.86×NDVI-33.29
	SPAD+NDRE	Y = 0.11×SPAD+23.43×NDRE-4.57	Y = -0.02×SPAD+39.07×NDRE-13.39	Y = 0.05×SPAD+17.46×NDRE+2.57
	SPAD+GNDVI	Y = 0.08×SPAD+41.62×GNDVI-21.04	Y = -0.01×SPAD+51.84×GNDVI-30.13	Y = 0.02×SPAD+25.24×GNDVI-5.57
	SPAD+NDVI+NDRE+GNDVI	Y = 0.02×SPAD-175.08×NDVI-109.34×NDRE+257.75×GNDVI+43.94	Y = -0.02×SPAD+2.26×NDVI+22.5×NDRE+21.92×GNDVI-22.43	Y = 0.07×SPAD+120.68×NDVI-19.4×NDRE-18.64×GNDVI-73.37
	NDVI+NDRE	Y = 86.96×NDVI+11.76×NDRE-74.35	Y = 29.35×NDVI+29.95×NDRE-35.64	Y = 108.48×NDVI-23.67×NDRE-70.56
	NDVI+GNDVI	Y = -123.27×NDVI+87.82×GNDVI+63.59	Y = -51.89×NDVI+65.44×GNDVI+6.84	Y = 118.17×NDVI-33.28×GNDVI-66.28
	NDRE+GNDVI	Y = -96.93×NDRE+186.8×GNDVI-72.7	Y = 19.62×NDRE+23.01×GNDVI-20.09	Y = -58.01×NDRE+96.82×GNDVI-28.72
	NDVI+NDRE+GNDVI	Y = -192.22×NDVI-110.42×NDRE+266.83×GNDVI+54.9	Y = -10.82×NDVI+17.28×NDRE+29.49×GNDVI-13.7	Y = 102.85×NDVI-28.86×NDRE+9.14×GNDVI-69.73
SER	SPAD	Y = 3.85 × e^(0.02×SPAD)^	Y = 4.34 × e^(0.02×SPAD)^	Y = 4.57 × e^(0.02×SPAD)^
	NDVI	Y = 0.00002 × e^(14.62×NDVI)^	Y = 0.000003 × e^(16.14×NDVI)^	Y = 0.11 × e^(5.47×NDVI)^
	NDRE	Y = 2.25 × e^(3.35×NDRE)^	Y = 0.99 × e^(3.71×NDRE)^	Y = 4.92 × e^(2.18×NDRE)^
	GNDVI	Y = 0.27 × e^(5.31×GNDVI)^	Y = 0.19 × e^(5.04×GNDVI)^	Y = 1.97 × e^(2.72×GNDVI)^
MER	SPAD+NDVI	Y = 0.0001 × e^(0.01×SPAD+11.83×NDVI)^	Y = 0.000003 × e^(-0.0003×SPAD+16.32×NDVI)^	Y = 0.13 × e^(0.001×SPAD+5.3×NDVI)^
	SPAD+NDRE	Y = 2 × e^(0.01×SPAD+2.77×NDRE)^	Y = 0.87 × e^(-0.002×SPAD+4.06×NDRE)^	Y = 4.64 × e^(0.01×SPAD+1.64×NDRE)^
	SPAD+GNDVI	Y = 0.31 × e^(0.01×SPAD+4.75×GNDVI)^	Y = 0.14 × e^(-0.002×SPAD+5.49×GNDVI)^	Y = 2.07 × e^(0.002×SPAD+2.5×GNDVI)^
	SPAD+NDVI+NDRE+GNDVI	Y = 15372.43 × e^(-0.001×SPAD-23.51×NDVI-10.65×NDRE+28.04×GNDVI)^	Y = 1.78 × e^(-0.002×SPAD-3.83×NDVI-0.27×NDRE+7.01×GNDVI)^	Y = 0.001 × e^(0.01×SPAD+11.02×NDVI-4.55×NDRE1.91×GNDVI)^
	NDVI+NDRE	Y = 0.01 × e^(6.3×NDVI+2×NDRE)^	Y = 0.01 × e^(5.38×NDVI+2.5×NDRE)^	Y = 0.001 × e^(12.14×NDVI-2.95×NDRE)^
	NDVI+GNDVI	Y = 17925.87 × e^(-15.84×NDVI+10.32×GNDVI)^	Y = 1.78 × e^(-3.28×NDVI+6.04×GNDVI)^	Y = 0.004 × e^(12.38×NDVI-3.64×GNDVI)^
	NDRE+GNDVI	Y = 0.003 × e^(-9.01×NDRE+18.13×GNDVI)^	Y = 0.21 × e^(0.26×NDRE+4.69×GNDVI)^	Y = 0.09 × e^(-8.04×NDRE+12.35×GNDVI)^
	NDVI+NDRE+GNDVI	Y = 7793.15 × e^(-22.45×NDVI-10.58×NDRE+27.48×GNDVI)^	Y = 5.32 × e^(-5.47×NDVI-0.92×NDRE+7.97×GNDVI)^	Y = 0.002 × e^(9.55×NDVI-5.34×NDRE+4.2×GNDVI)^

^a^SLR is simple linear regression; MLR is multiple linear regression; SER is simple exponential regression; MER is multiple exponential regression

For the single linear and exponential regression models (i.e., SLR and SER), NDRE and NDVI provided better model performance at the R3 stage (61 DAE) than either at the early (V6) or the mature stage (Fig 6 and Table 6). The weaker yield estimations during earlier flights (V6 stage) are consistent with previous work by Shajahan et al. [57] who found low yield estimates while evaluating six VIs in corn that received N side-dress treatments. Furthermore, the model trait combination with only VIs exhibited good performance (R^2^ > 0.73), except for the early growing season (V6). A combination of NDVI and NDRE traits also showed reliable model performance (R^2^ = 0.81 and 0.85 for MLR and MER, respectively) (Fig 6). Torino et al. [58] and Paiao et al. [56] found that NDVI provided less accuracy in predicting grain yield than NDRE regardless of crop development stage. Contrastingly, in our study, NDVI was the best univariate indicator for corn grain yield prediction. The performance of NDVI improved when combined with NDRE and GNDVI. Compared to the morphological traits, the three VIs were more effective for grain yield prediction, as well as the combination of multiple predictors in the model.

We note that the SPAD provided a better yield prediction compared to other morphological trait measurements in this study, and it is a reliable predictor for grain yield prediction by capturing N status in the leaves under different N rates. As a single predictor, SPAD was least effective at predicting yield (R^2^ = 0.18–0.62 for SLR and R^2^ = 0.2–0.65 for SER). The SPAD-only models performed best at the R3 stage and were least effective at V6 (Fig 6). These findings align with Lindsey et al. [59] who also reported a strengthening in correlations between measured corn grain yield and SPAD and concluded that SPAD measurements at R1 to R2 stage (R^2^ = 0.75) predicted corn grain yields better than SPAD measurements at V6 stage (R^2^ = 0.23). Similarly, Paiao et al. [56] reported that the SPAD measurement at V8 to R1 (R^2^ = 0.85 with linear regression model) improved corn yield prediction compared to SPAD measurement at V4 stage (R^2^ = 0.65 with linear regression model). In addition, SPAD used for the combination of MLR and MER resulted in improved grain yield estimation (R^2^ = 0.28–0.82 MLR and R^2^ = 0.34–0.86 for MER). Regressions combining SPAD and VIs improved prediction (R^2^ > 0.69) after the R3 stage.

Compared to the multivariate models, the univariate SLR and SER regression models were more effective for grain yield prediction. During the R3 and mature stages, slightly better R^2^ was obtained when multiple predictors were combined into the grain yield prediction model, with the outcome being that R^2^ was greater than 0.69 (Fig 6). Similar studies have reported that combining appropriate variables instead of using one variable alone more accurately predicts corn grain yield [47, 54, 60]. For example, Freeman et al. [60] found that the combination of NDVI and plant height indices were able to provide a higher correlation with corn forage yield than NDVI and plant height separately (R^2^ = 0.62, 0.52, and 0.59, respectively). Liu and Wiatrak [47] showed that combining plant height, NDVI at R1 growth stage, and LAI at R1 growth stage explained 71% of variability in corn grain yield. According to Edalat et al. [54], the combination of leaf nitrogen, SPAD, and NDVI in the regression equation could be considered as a potential tool to predict corn grain yield.

Model performance was consistent or slightly improved after 5-fold cross-validation (Table 7). The models developed using traits from the late season (mature) showed better performance, including low RMSE and MAE, than the early and middle growing seasons (V6 and R3). We found that the exponential regression model was suited to predict grain yield using the traits from the early sampling season (V6). In contrast, the linear regression model using mid and late season traits (R3 and mature) had better grain yield prediction than the exponential regression model. The best grain yield prediction model after 5-fold cross-validation was a multiple linear regression model using the combination of NDVI and GNDVI (RMSE = 0.97, MAE = 0.78, R^2^ = 0.84).

**Table 7:** Corn grain yield estimation results in 5-fold cross-validation based on simple linear regression (SLR), multiple linear regression (MLR), simple exponential regression (SER), and multiple exponential regression (MER) model. The predictors were collected in the early (V6), middle (R3), and late (mature) corn growth stages.

Model^a^	Predictor(s)	Early (V6)^b^			Middle (R3)^b^			Late (Mature)^b^		
		RMSE (Mg ha^-1^)	MAE (Mg ha^-1^)	R^2^	RMSE (Mg ha^-1^)	MAE (Mg ha^-1^)	R^2^	RMSE (Mg ha^-1^)	MAE (Mg ha^-1^)	R^2^
SLR	SPAD	2.23	1.75	0.25	1.52	1.26	0.71	1.51	1.31	0.59
	NDVI	2.12	1.71	0.34	1.20	0.94	0.70	1.06	0.85	0.81
	NDRE	2.11	1.77	0.31	1.19	0.91	0.72	1.34	1.16	0.72
	GNDVI	2.00	1.65	0.38	1.18	0.91	0.71	1.25	1.06	0.75
MLR	SPAD+NDVI	2.11	1.70	0.35	1.24	0.96	0.69	1.13	0.90	0.78
	SPAD+NDRE	2.16	1.80	0.29	1.26	0.95	0.69	1.38	1.21	0.69
	SPAD+GNDVI	2.06	1.70	0.33	1.23	0.93	0.70	1.32	1.12	0.72
	SPAD+NDVI+NDRE+GNDVI	2.07	1.67	0.34	1.33	1.01	0.67	1.02	0.81	0.80
	NDVI+NDRE	2.15	1.74	0.31	1.26	0.97	0.70	0.99	0.82	0.85
	NDVI+GNDVI	2.06	1.67	0.33	1.23	0.96	0.70	0.97	0.78	0.84
	NDRE+GNDVI	1.89	1.48	0.47	1.25	0.97	0.70	1.23	0.93	0.74
	NDVI+NDRE+GNDVI	1.90	1.49	0.46	1.27	0.99	0.69	1.07	0.86	0.81
SER	SPAD	1.24	1.18	0.30	1.15	1.13	0.70	1.16	1.14	0.67
	NDVI	1.22	1.16	0.47	1.12	1.10	0.78	1.11	1.09	0.80
	NDRE	1.22	1.17	0.46	1.12	1.10	0.76	1.14	1.13	0.70
	GNDVI	1.21	1.16	0.48	1.11	1.10	0.77	1.13	1.11	0.73
MER	SPAD+NDVI	1.23	1.17	0.46	1.12	1.10	0.76	1.12	1.10	0.77
	SPAD+NDRE	1.23	1.17	0.44	1.12	1.10	0.74	1.15	1.13	0.69
	SPAD+GNDVI	1.22	1.17	0.47	1.12	1.10	0.75	1.14	1.12	0.71
	SPAD+NDVI+NDRE+GNDVI	1.20	1.17	0.48	1.13	1.11	0.71	1.10	1.08	0.79
	NDVI+NDRE	1.22	1.16	0.47	1.12	1.10	0.75	1.09	1.07	0.85
	NDVI+GNDVI	1.21	1.17	0.48	1.12	1.10	0.74	1.10	1.08	0.83
	NDRE+GNDVI	1.20	1.15	0.48	1.12	1.11	0.74	1.11	1.09	0.73
	NDVI+NDRE+GNDVI	1.19	1.15	0.48	1.13	1.11	0.72	1.10	1.08	0.78

^a^SLR is simple linear regression; MLR is multiple linear regression; SER is simple exponential regression; MER is multiple exponential regression

^b^RMSE is root mean square error; MAE is mean absolute error

Early-season yield predictions can help producers notice yield-limiting factors and make informed agronomic decisions that include interventions like side dressing, additional fertilization, and irrigation to improve plant health and ultimately increase yield at harvest. While the crop is still in the field, late-season yield predictions offer producers practical insights that can help them make more effective marketing decisions and more precise insurance estimates [61]. This study validates that in-season remote-sensed imageries can effectively differentiate plant health based on fertilizer treatments. Importantly, the vegetation indices described spatial variations in plant health and could provide information for specific areas that require additional interventions. These results hold great potential for refining models that facilitate spatial-specific fertilizer prescriptions throughout the growing season. Future research should explore the possibility of including additional data (such as various environments, the wide N ranges, the comprehensive understanding of slow-release fertilizers, and diverse vegetation indices) to improve prediction accuracy further and make more agronomically insightful discoveries.

## Conclusions

As remote sensed vegetation indices and ground truthing data are common variables to predict corn yield, it is important to understand how monitoring timing, morphological traits, and variable selection affect the model prediction improvement. In this study, both linear and exponential regression models were adopted to estimate corn grain yield using single and multi-variable approaches which drew from the morphological and remote sensed traits. The late growing season (after VT stage) SPAD measurements performed better than height in grain yield prediction. In addition, Vegetation Indices (NDVI, NDRE, and GNDVI) were preferable to morphological trait measurements in grain prediction. The VIs collected in the earliest season (V6) derived low accuracy in grain prediction (R^2^ < 0.51), but the model improved when GNDVI and NDRE after the R3 corn growth stage were used as a single variable. Of the VIs, NDVI measured late in the season (mature) showed a better prediction of grain yield using a linear model. The yield prediction can however be improved by combining NDVI and GNDVI using multiple linear regression. In addition, the VIs can detect in-season variability among various N application treatments, especially different N levels within CRF treatment. Therefore, VIs collected from UAV monitoring can be used for complex experimental conditions to detect plant health differences under various treatments and effectively manage farm operations and N prescriptions. However, future studies require building a machine-learning algorithm for an N application prescription tool based on the wide N ranges of each N source and a better understanding of CRF fertilizer.

## Supporting information

S1 Data (CSV)
